# Mesoporous inorganic salts with crystal defects: unusual catalysts and catalyst supports†
†Electronic supplementary information (ESI) available: Scheme S1 contains reaction equation, Fig. S1–S7 contain solubility test, XRD, SEM, TEM, micropore size distribution and reaction conversion. See DOI: 10.1039/c4sc03736g



**DOI:** 10.1039/c4sc03736g

**Published:** 2015-01-06

**Authors:** Xinchen Kang, Wenting Shang, Qinggong Zhu, Jianling Zhang, Tao Jiang, Buxing Han, Zhonghua Wu, Zhihong Li, Xueqing Xing

**Affiliations:** a Beijing National Laboratory for Molecular Sciences , Key Laboratory of Colloid and Interface and Thermodynamics , Institute of Chemistry , Chinese Academy of Sciences , Beijing 100190 , China . Email: jiangt@iccas.ac.cn ; Email: hanbx@iccas.ac.cn; b Beijing Synchrotron Radiation Facility , Institute of High Energy Physics , Chinese Academy of Sciences , Beijing 100049 , China

## Abstract

We proposed a strategy to synthesize mesoporous inorganic salt particles using the special properties of ionic liquid (IL) mixtures, and hollow mesoporous LaF_3_, NdF_3_, and YF_3_ particles were synthesized and characterized using different techniques. The size of the mesopores in the salt particles was about 4 nm, and the materials were full of crystal defects. The LaF_3_, NdF_3_ and YF_3_ particles were used as the catalysts for the cyanosilylation reaction of benzaldehyde using trimethylsilyl cyanide, and Ru/LaF_3_ and Ru/NdF_3_, in which Ru nanocatalysts were supported on the LaF_3_ and NdF_3_ particles with mesopores, were used to catalyze hydrogenations of benzene to cyclohexane and levulinic acid (LA) to γ-valerolactone (GVL). It was discovered that the activities of these catalysts were unprecedentedly high for these reactions. Detailed study showed that both the crystal defects and the mesopores in the salt particles played crucial roles for the extremely high catalytic activity.

## Abstract 2

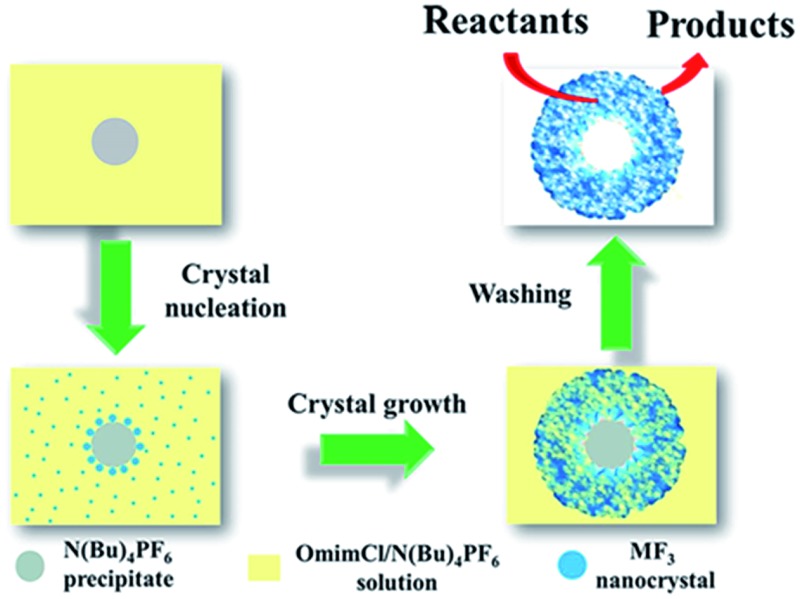
Mesoporous LaF_3_, NdF_3_, and YF_3_ particles with crystal defects, which are excellent catalysts and catalyst supports, have been synthesized successfully.

## Introduction

Porous materials have wide-ranging applications due to their admirable and intriguing structures. Common porous materials include porous carbon materials,^1^ porous polymers,^2^ zeolite materials,^3^ metal–organic frameworks (MOFs),^4^ and covalent organic frameworks (COFs),^5^ and so on. Porous materials have a variety of applications or potential applications, such as catalysis,^6^ gas separation, capture and storage,^7^ and electrode materials.^8^ Inorganic salts are composed of cations and anions of small size. The strong interaction among the ions leads to a close-packed structure of the salts, which is more feasible to form a perfect crystal. The reports about preparation of porous salts are very limited. Cong *et al.* synthesized NaCl and KCl with macropores (380 nm in diameter) using copolymer colloids as the template.^9^ Chu *et al.* prepared porous FeF_3_ particles consisting of small nanoparticles through two steps: Fe(OH)_3_ synthesis and FeF_3_ fabrication.^10^

Ionic liquids (ILs) have received much interest due to their special properties such as negligible vapor pressure, good thermal stability, high conductivity, and strong solvent power for both organic and inorganic substances.^11^ Especially, ILs have a designable nature.^12^ These unique properties make them have great potential in applications in different fields, such as material synthesis,^13^ chemical reactions,^14^ extraction and fractionation,^15^ and gas absorption.^16^

Catalysis is quite prevalent in chemical reactions and chemical industry.^17^ Supported metal catalysts are widely used because the metal components are active for various reactions.^18^ It is well known that for supported catalysts, catalyst supports often have great influence on the activity, selectivity, and stability of the catalysts.^19^ Mesoporous inorganic salt particles may have many advantages in catalysis which are not known because their synthesis is challenging.

Herein, we proposed a strategy to synthesize porous inorganic salts using IL–organic salt mixtures. LaF_3_, NdF_3_, and YF_3_ particles with mesopores were successfully synthesized. As examples of applications, the mesoporous salt particles were used both as the catalysts directly and supports of Ru nanocatalysts. It was discovered that the salts and the salt supported catalysts exhibited unprecedented activity for different reactions. Systematic study indicated that both the crystal defects and mesopores contributed significantly to the very high catalytic activity. To the best our knowledge, this is the first work to use mesoporous inorganic salts as catalysts and catalyst supports.

## Results and discussion

We synthesized the inorganic salts in tetrabutylammonium hexafluorophosphate (N(Bu)_4_PF_6_) and IL 1-octyl-3-methylimidazolium chloride (OmimCl) mixtures. N(Bu)_4_PF_6_ is an organic salt with a melting point of 246–247 °C.^20^ OmimCl is liquid at room temperature. Fig. S1† shows the solubility of N(Bu)_4_PF_6_ (*x*_2_, in mole fraction) in OmimCl determined in this work. The solubility of N(Bu)_4_PF_6_ in OmimCl decreases with decreasing temperature. Therefore, some N(Bu)_4_PF_6_ will precipitate as its content in the OmimCl–N(Bu)_4_PF_6_ mixture exceeds the solubility (Fig. S1†).

**Fig. 1:**
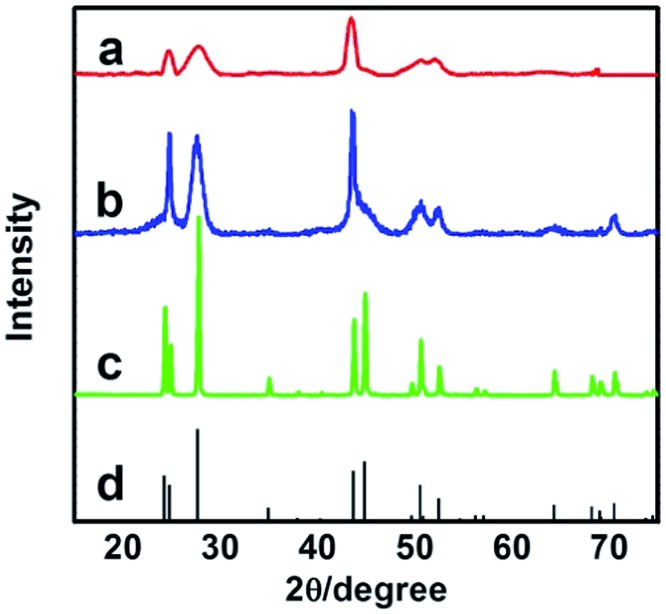
XRD patterns of LaF_3_: (a) LaF_3_ synthesized at *x*_2_ = 0.5; (b) LaF_3_ synthesized at *x*_2_ = 0.33; (c) commercial LaF_3_; and (d) standard pattern.

**Fig. 2:**
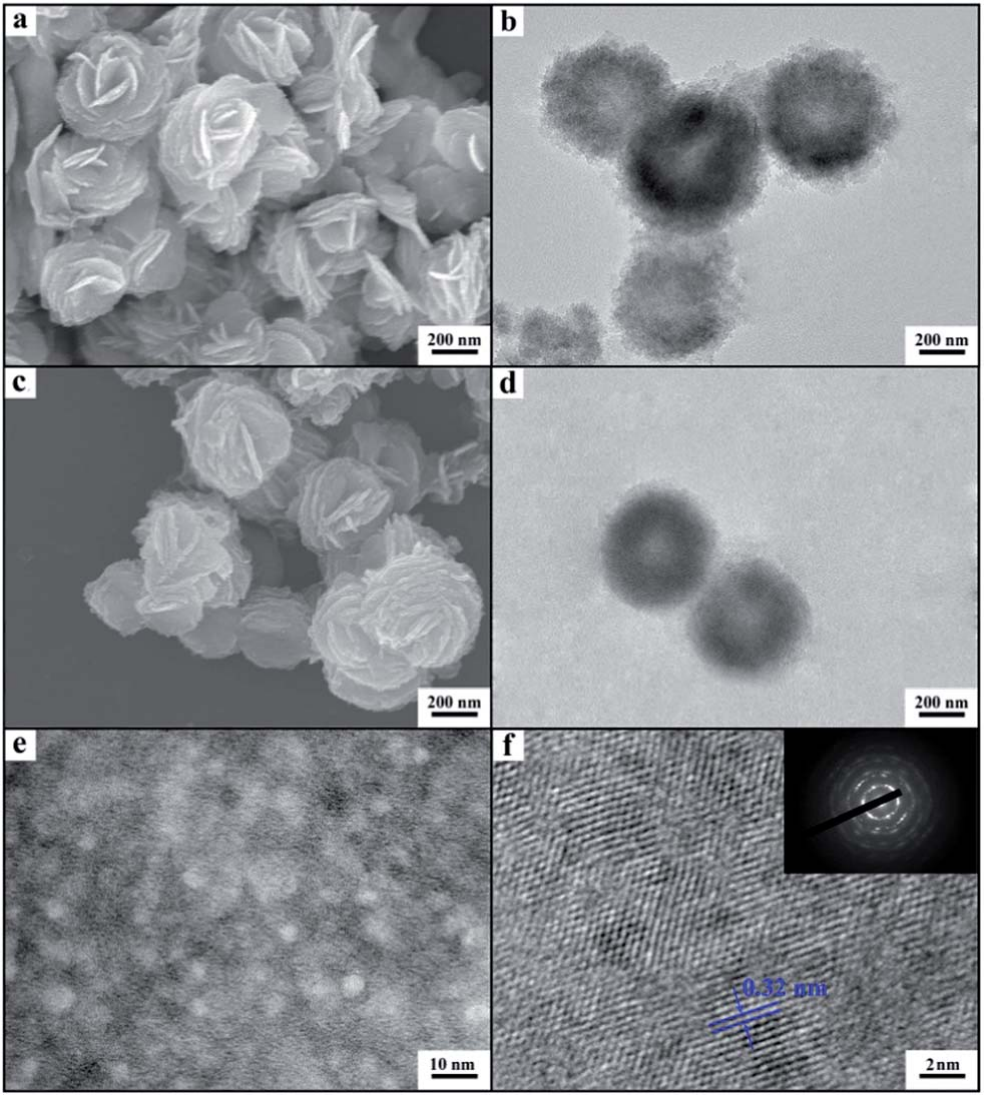
SEM and TEM images of mesoporous LaF_3_ synthesized in the OmimCl–N(Bu)_4_PF_6_ mixtures: (a) SEM image of the LaF_3_ synthesized at *x*_2_ = 0.5; (b) TEM image of the LaF_3_ synthesized at *x*_2_ = 0.5; (c) SEM image of the LaF_3_ synthesized at *x*_2_ = 0.33; (d) TEM image of the LaF_3_ synthesized at *x*_2_ = 0.33; (e) HRTEM image to show the pores on the walls of the LaF_3_ synthesized at *x*_2_ = 0.5; and (f) HRTEM image to show the crystal structure and the SAED pattern (the inset pattern) of the LaF_3_ synthesized at *x*_2_ = 0.5.

We synthesized LaF_3_ in OmimCl–N(Bu)_4_PF_6_ mixtures at *x*_2_ (mole fraction of N(Bu)_4_PF_6_ in OmimCl–N(Bu)_4_PF_6_ mixture) of 0.50 and 0.33 using La(NO_3_)_3_ as the metal precursor. Some precipitated N(Bu)_4_PF_6_ particles existed in the OmimCl–N(Bu)_4_PF_6_ mixtures at these conditions. The LaF_3_ was generated from La(NO_3_)_3_ and N(Bu)_4_PF_6_, and the reaction equation is shown in Scheme S1.† ^21^ The X-ray diffraction (XRD) patterns of the as-prepared materials demonstrate clearly the formation of LaF_3_ (Fig. 1). The half-peak width in the XRD patterns was much larger than the commercial LaF_3_ (Fig. 1), indicating the poor crystallinity and existence of significant amounts of crystal defects in the LaF_3_ prepared in this work. The scanning electron microscopy (SEM) and transmission electron microscopy (TEM) images of the LaF_3_ materials are given in Fig. 2. The size of the mesopores in the particles was about 4 nm (Fig. 2e), which are intracrystal pores. The size of the particles was about 400 nm and each particle had a hollow core of about 80 nm.

**Fig. 3:**
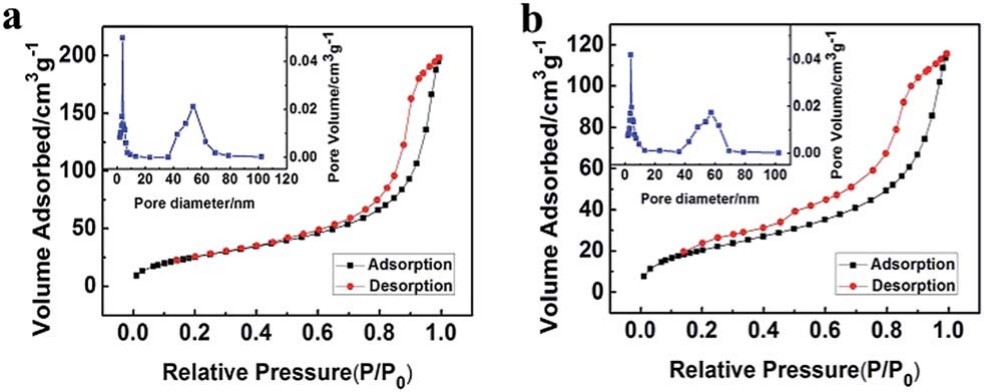
N_2_ adsorption/desorption isotherms and pore size distribution (the insets) of the LaF_3_ particles synthesized at *x*_2_ = 0.5 (a) and 0.33 (b).

The porous LaF_3_ particles prepared were further characterized using the N_2_ adsorption/desorption method, and the adsorption/desorption isotherms and the pore size distributions of the LaF_3_ particles are given in Fig. 3. The pore size distribution curve of the LaF_3_ particles prepared had two peaks, which centered at about 4 nm and 55 nm, corresponding to the mesopores in the walls and hollow cores of the particles, respectively. The size of the pores determined by the N_2_ adsorption/desorption method was roughly consistent with that observed from the TEM images (Fig. 2). The surface areas obtained from the BET (Brunauer, Emmett, and Teller) method and the pore volumes calculated from the Barrett–Joyner–Halenda method are presented in Table 1. The results further demonstrated the mesoporous character.

**Table 1:** The BET surface area (*S*) and total pore volume (*V*) of different materials

Entry	Samples	*S* (m^2^ g^–1^)	*V* (cm^3^ g^–1^)
1	LaF_3_ ^*a*^	97.9	0.32
2	LaF_3_ ^*b*^	77.6	0.26
3	LaF_3_ ^*c*^	59.1	0.16
4	NdF_3_ ^*d*^	98.7	0.34
5	YF_3_ ^*e*^	95.7	0.28
6	LaF_3_ ^*f*^	0.48	0.002
7	La-MOF^*g*^	28.5	0.05

^*a*^Porous LaF_3_ synthesized at *x*_2_ = 0.5 (Fig. 2a and b).

^*b*^Porous LaF_3_ synthesized at *x*_2_ = 0.33 (Fig. 2c and d).

^*c*^Porous LaF_3_ synthesized at *x*_2_ = 0.17 (Fig. S3a and b).

^*d*^Porous NdF_3_ synthesized at *x*_2_ = 0.5 (Fig. 8a and b).

^*e*^Porous YF_3_ synthesized at *x*_2_ = 0.5 (Fig. 8c and d).

^*f*^Commercial LaF_3_ (Fig. S4a and b).

^*g*^La-MOF prepared in this work (Fig. S4c and d).

**Fig. 4:**
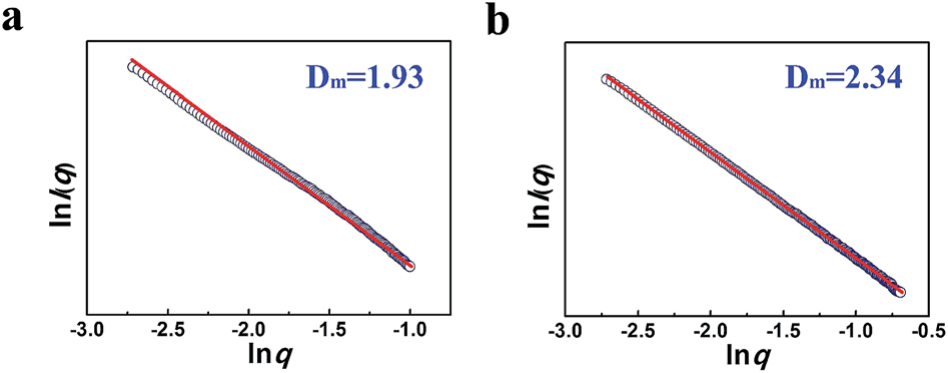
Mass fractal dimension (*D*_m_) from SAXS curves of the as-prepared LaF_3_ materials synthesized at *x*_2_ = 0.5 (a), 0.33 (b).

The compactness of the materials can be characterized by the small angle X-ray scattering (SAXS) technique *via* analyzing the fractal dimension *D*.^22^ In this work we conducted a SAXS study (Fig. 4). According to the SAXS data, it can be known that the mass fractal existed in the LaF_3_ materials, indicating that the materials had a loose structure with lots of crystal defects,^22^ which agrees with the fact that the salt particles had poor crystallinity as shown by the XRD patterns (Fig. 1). Moreover, the LaF_3_ synthesized had plenty of micropores ranging from 0.5 nm to 2 nm, and the pore size distribution is shown in Fig. S2.† The surface area of the micropores was 1.0 m^2^ g^–1^, which further confirms the existence of crystal defects in the LaF_3_ materials. The crystal defects in the LaF_3_ materials originate mainly from the mesopores, edges, and tips in the salts.

**Fig. 5:**
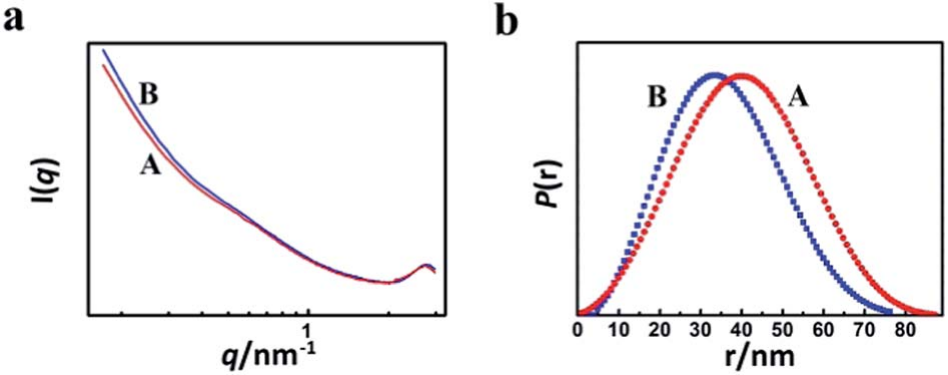
SAXS curves (a) and normalized pair-distance distribution function curves (b) of the OmimCl–N(Bu)_4_PF_6_ mixtures at *x*_2_ = 0.5 (A) and 0.33 (B).

In order to study the reason for the formation of the hollow cores and mesopores in the LaF_3_ particles synthesized, we studied the size and shape of the precipitated N(Bu)_4_PF_6_ particles in the OmimCl–N(Bu)_4_PF_6_ mixtures using the SAXS technique, which is commonly used for characterizing the size and shape of particles dispersed in liquids.^23^ The SAXS curves of the mixtures with different compositions are presented in Fig. 5a. The data in the small-angle region (*q* < 1) were used to obtain the size of the precipitated N(Bu)_4_PF_6_ particles. The generalized indirect Fourier transformation gives the pair-distance distribution function, *p*(r), which is shown in Fig. 5b. The curves were nearly symmetric, suggesting that the precipitated N(Bu)_4_PF_6_ particles in the OmimCl–N(Bu)_4_PF_6_ mixtures were spherical.^23^ The size of the N(Bu)_4_PF_6_ particles calculated from the SAXS data agreed roughly with that of the hollow cores of the LaF_3_ particles shown in Fig. 2. SAXS is also a useful technique to study the microstructure of ILs. It has highlighted the existence of the nano-scale aggregates, and the size can be estimated using Bragg's diffraction law *d* = 2π/*q*.^24^ The size of the IL aggregates calculated using this method was 3 nm. These could act as the templates to form the mesopores of the LaF_3_ particles.

**Fig. 6:**
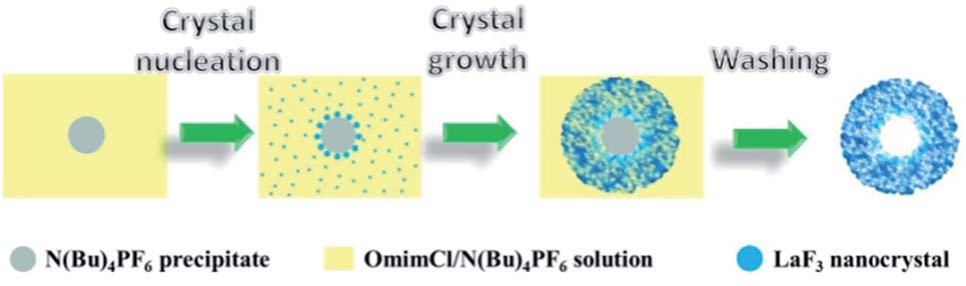
The schematic diagram for the formation mechanism of mesoporous LaF_3_ particles with a hollow core.

On the basis of the experimental results we can discuss the formation processes of the mesoporous particles, which are shown schematically in Fig. 6. In the OmimCl–N(Bu)_4_PF_6_ mixtures, some N(Bu)_4_PF_6_ exists as the precipitated particles at *x*_2_ = 0.5 and 0.33, and the rest of the N(Bu)_4_PF_6_ is dissolved in the solution, as discussed above. The La(NO_3_)_3_ reacts with the N(Bu)_4_PF_6_ particles at the surface to generate LaF_3_. At the same time, the La(NO_3_)_3_ also reacts with the N(Bu)_4_PF_6_ in the solution. The LaF_3_ generated in the solution aggregates with the LaF_3_ nanocrystals on the surface of the N(Bu)_4_PF_6_ particles. Some IL aggregates are entrained when the LaF_3_ particles aggregate around the N(Bu)_4_PF_6_ particles. The regular hollow LaF_3_ particles with mesopores are formed after removing the OmimCl and N(Bu)_4_PF_6_ by washing with acetone. In this process, the N(Bu)_4_PF_6_ particles and the entrained IL aggregates act as the templates for the core and the mesopores, respectively (Fig. 2 and 6). In order to get the evidence that the N(Bu)_4_PF_6_ particles act as the template for the formation of the hollow core, we also synthesized the LaF_3_ particles in the OmimCl–N(Bu)_4_PF_6_ mixture at *x*_2_ = 0.17, and the SEM and TEM images, XRD patterns, and N_2_ adsorption/desorption isotherms are given in Fig. S3.† In this case, all the N(Bu)_4_PF_6_ dissolves in OmimCl (Fig. S1†). La(NO_3_)_3_ reacts with the N(Bu)_4_PF_6_ in the solution due to the absence of the precipitated N(Bu)_4_PF_6_ particles, and the generated LaF_3_ aggregates arbitrarily and irregular LaF_3_ particles are formed (Fig. S3†). The particles with mesopores were also obtained after removing the entrained OmimCl and N(Bu)_4_PF_6_, but there was no hollow core (Fig. S3†). The results support the argument that the precipitated N(Bu)_4_PF_6_ particles act as the template for the formation of the hollow core of the salt particles at higher N(Bu)_4_PF_6_ concentrations (*x*_2_ = 0.5 and 0.33).

**Fig. 7:**
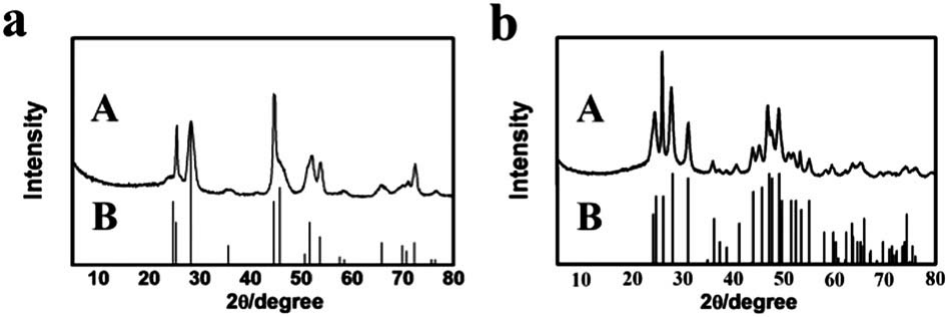
XRD patterns of the as-synthesized fluoride salts (A) synthesized at *x*_2_ = 0.5 and the corresponding standard patterns (B): (a) NdF_3_; and (b) YF_3_.

**Fig. 8:**
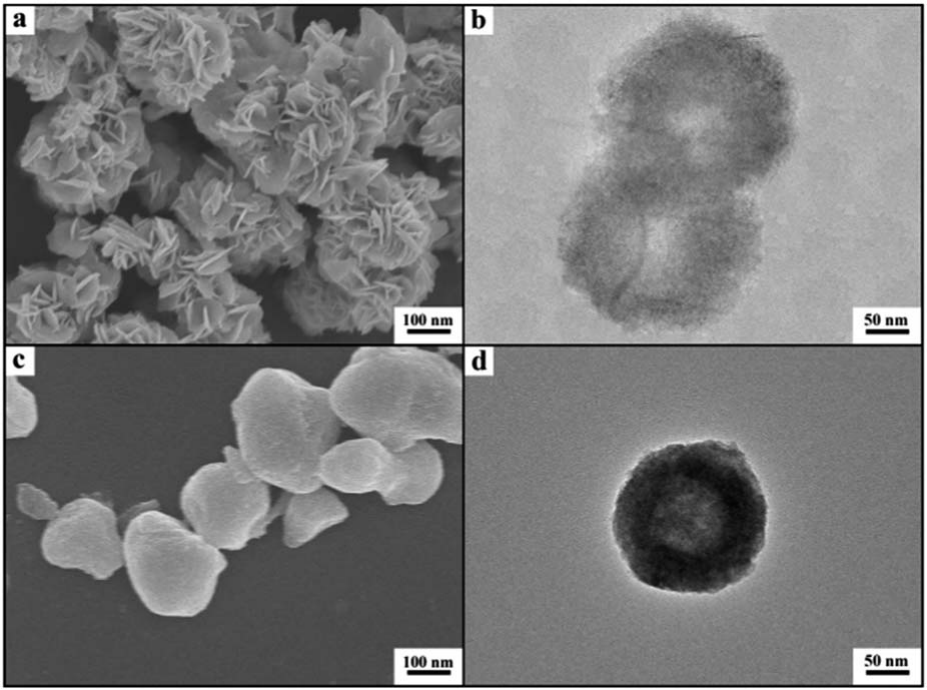
SEM and TEM images of NdF_3_ (a and b) and YF_3_ (c and d) materials synthesized at *x*_2_ = 0.5.

We also synthesized NdF_3_ and YF_3_ particles in the OmimCl–N(Bu)_4_PF_6_ mixture of *x*_2_ = 0.50 using this method. The XRD patterns, SEM and TEM images are given in Fig. 7 and 8, respectively. The porosity properties determined using the N_2_ adsorption/desorption method are given in Table 1. The XRD patterns confirmed the formation of NdF_3_ and YF_3_ (Fig. 7). All the characterizations indicate that the morphology, structure, and porosity properties of the NdF_3_ and YF_3_ particles were similar to those of LaF_3_ prepared, which indicates that the strategy proposed in this work is versatile.

The mesoporous salt particles may find wide-ranging applications in different fields. They are very stable inorganic salts and are not soluble in water and common organic solvents, which are excellent characteristics of heterogeneous catalysts and catalyst supports. As examples of applications, we used the salt particles both as a catalyst directly and as the support of metal nanocatalysts for different reactions.

**Scheme 1:**
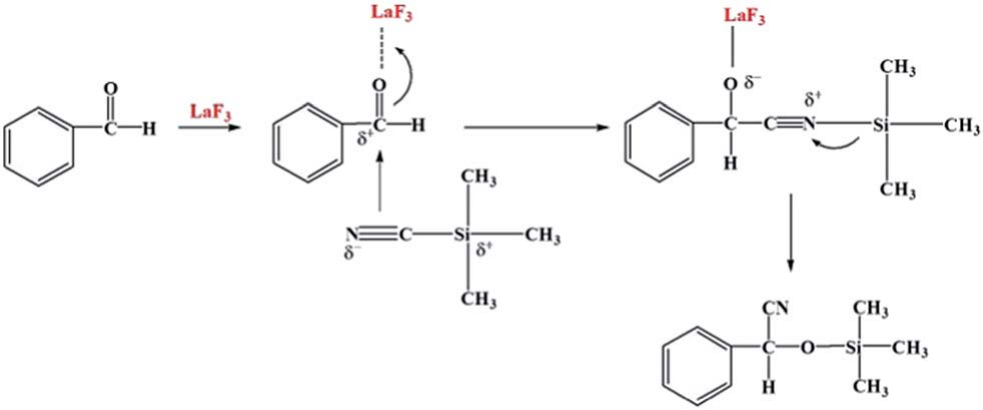
The reaction mechanism for cyanosilylation of benzaldehyde to cyanohydrin.

Cyanosilylation of carbonyl compounds provides a convenient route to produce cyanohydrins,^25^ which are key intermediates in the synthesis of fine chemicals and pharmaceuticals.^26^ The mesoporous LaF_3_ shown in Fig. 2a and b was used as a heterogeneous catalyst in the cyanosilylation reaction of benzaldehyde and trimethylsilyl cyanide (TMSCN) to produce cyanohydrin, and the results are given in Table 2. Surprisingly, the LaF_3_ particles with mesopores were extremely active and selective for the reaction (entry 1). In addition, the mesoporous NdF_3_ (Fig. 8a and b) and YF_3_ (Fig. 8c and d) had similar activity for this reaction (entries 2–3). We also studied the activity of commercial LaF_3_ for this reaction (entry 4), and the activity was much lower (Fig. S4a and b† and Table 1). It has been reported that La metal–organic framework (La-MOF) was an efficient catalyst for this reaction.^27^ In this work, we synthesized the La-BTC MOF (La-MOF hereafter). The as-prepared La-MOF was characterized by SEM and TEM (Fig. S4c and d†), XRD (Fig. S5†), and N_2_ adsorption/desorption (Table 1) methods. The XRD pattern was the same as that reported previously.^28^ The La-MOF was also used to catalyze the reaction, and it was demonstrated that the activity of the mesoporous LaF_3_ particles was much higher than the La-MOF (entries 1 and 5) for the reaction. In order to study the effect of the defects in the mesoporous LaF_3_, NdF_3_ and YF_3_ particles on the reaction, we calculated the turnover frequencies (TOFs) per unit surface area of all the catalysts in Table 2, which were calculated from the reaction data in Table 2 and the surface area data in Table 1. Surprisingly, the TOFs per unit surface area of the as-prepared mesoporous LaF_3_, NdF_3_ and YF_3_ were much higher than the commercial LaF_3_ that had no pores, and the La-MOF. It is well known that the mass transfer in mesopores is poorer than that at the surface of nonporous materials. Therefore, it can be concluded that the crystal defects of the mesoporous LaF_3_, NdF_3_ and YF_3_ contributed significantly to their extremely high activity. The reaction mechanism is shown in Scheme 1. The reaction is catalyzed by Lewis acid.^27^ The crystal defects result in Lewis acid sites,^29^ which enhances the acidity and is favourable for accelerating the reaction. The commercial LaF_3_ has limited Lewis acid sites due to the high crystallinity. Thus, both the mesopores and the crystal defects in the salt particles played crucial roles for the very high catalytic activity.

**Table 2:** Cyanosilylation of benzaldehyde using TMSCN^*a*^ ^,^ ^*i*^

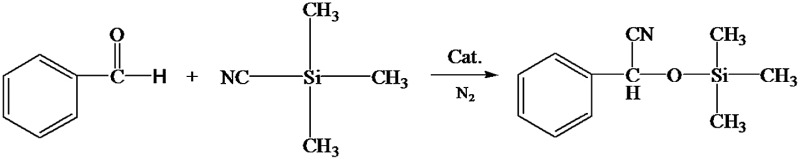					
Entry	Catalysts	*t* (min)	*C* (%)	TOF_1_ ^*b*^ (h^–1^)	TOF_2_ ^*c*^ (g h^–1^ m^–2^)
1	LaF_3_ ^*d*^	1	100	1200	12.3
2	NdF_3_ ^*e*^	1	100	1200	12.2
3	YF_3_ ^*f*^	1	100	1200	12.5
4	LaF_3_ ^*g*^	10	2.2	2.6	5.4
5	La-MOF^*h*^	10	6.4	7.7	0.27

^*a*^Reaction conditions: 2 mL benzaldehyde, 5 mol% catalyst, benzaldehyde : TMSCN molar ratio of 1 : 1.5, 50 °C, solvent free.

^*b*^TOF_1_ is the turnover frequency, which was calculated as moles of converted benzaldehyde per mole of catalyst per hour.

^*c*^TOF_2_ is the turnover frequency, which was calculated as moles of converted benzaldehyde per mole of catalyst per hour per unit area, and surface area data determined in this work (Table 1) were used in the calculation.

^*d*^Porous LaF_3_ (Fig. 2a and b).

^*e*^Porous NdF_3_ (Fig. 8a and b).

^*f*^Porous YF_3_ (Fig. 8c and d).

^*g*^Commercial LaF_3_ (Fig. S4a and b).

^*h*^La-MOF prepared in this work (Fig. S4c and d). The quantitative analysis of the product was carried out using GC.

^*i*^ *C* = conversion of benzaldehyde.

We also used the mesoporous LaF_3_ and NdF_3_ particles (Fig. 2a and b and 8a and b) as catalyst supports. The Ru/LaF_3_ and Ru/NdF_3_ were synthesized using water as the solvent, RuCl_3_ as the precursor, and NaBH_4_ as the reductant. The loading of Ru in the catalysts was 1.0 wt% as determined using the ICP-AES (VISTA-MPX) method. The TEM image of the Ru/LaF_3_ and the size distribution of the Ru particles are shown in Fig. S6.† The Ru nanoparticles of about 1.2 nm were immobilized uniformly on the supports. The Ru nanoparticles in the Ru/NdF_3_ had a similar size and size distribution.

**Scheme 2:**
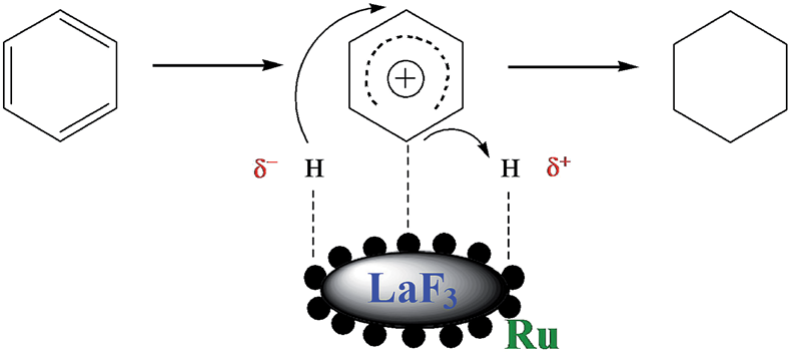
The reaction mechanism for benzene hydrogenation to cyclohexane.

**Fig. 9:**
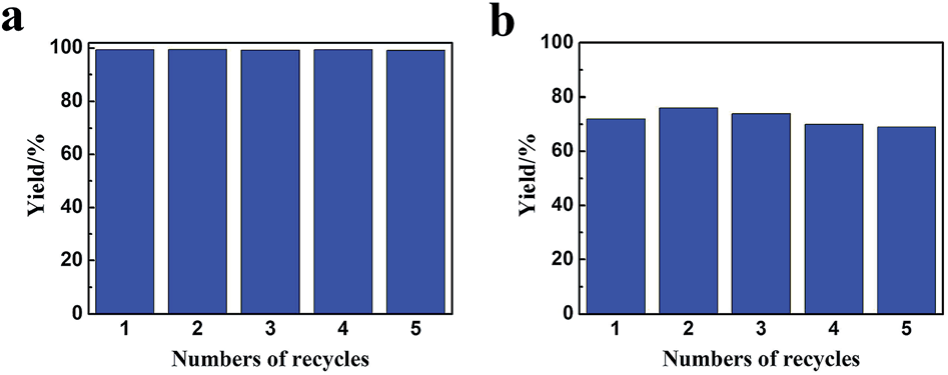
The reusability of the Ru/LaF_3_ catalyst for benzene hydrogenation to cyclohexane at 50 °C, 0.22 h (a) and 25 °C, 0.5 h (b). The other conditions were the same as in Table 1.

Benzene hydrogenation to cyclohexane is a very important reaction in chemical industry.^30^ We conducted the reaction using the Ru/LaF_3_, Ru/NdF_3_, and commercial Ru/C (TEM and SEM images are given in Fig. S6b and c†) as the catalysts at the same conditions, and the results are shown in Table 3. The catalytic activity of the Ru/LaF_3_ and Ru/NdF_3_ was amazingly high compared with that of the commercial Ru/C. It is obvious that the mesoporous morphology of the LaF_3_ and NdF_3_ particles played an important role in the reaction. It has also been reported that Lewis acids can activate aromatic rings.^31^ LaF_3_ and NdF_3_, which are Lewis acids, activated the benzene ring and the Ru nanoparticles activated the H_2_, which made the reaction more feasible to take place (Scheme 2). The crystal defects in the LaF_3_ and NdF_3_ lead to the existence of partial charge, which enhances the Lewis acidity and therefore the crystal defects can promote the reaction. The reusability of the Ru/LaF_3_ catalyst was also studied. It was shown that the catalyst could be reused at least five times without reducing the activity, as shown in Fig. 9.

**Table 3:** Catalytic performances of different catalysts for benzene hydrogenation to cyclohexane^*a*^ ^,^ ^*f*^

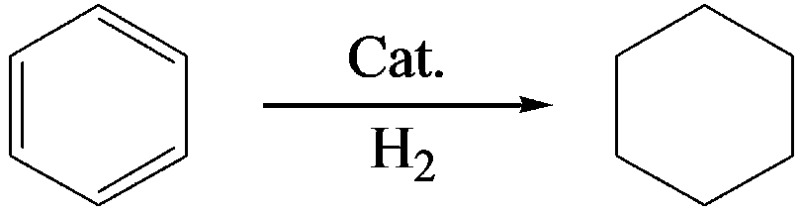					
Entry	Catalysts	*t* (h)	*T* (°C)	*Y* (%)	TOF^*b*^ (h^–1^)
1	Ru/LaF_3_ ^*c*^	0.22	50	>99	11 364
2	Ru/LaF_3_ ^*c*^	0.77	25	>99	3247
3	Ru/NdF_3_ ^*d*^	0.33	50	>99	7576
4	Ru/C^*e*^	8	50	95.5	298

^*a*^Reaction conditions: 2 mL benzene, benzene/Ru (mol mol^–1^) = 2500, 4 MPa H_2_.

^*b*^TOF was calculated as moles of converted benzene per mole of Ru per hour.

^*c*^Porous LaF_3_ (Fig. 2a and b) as support.

^*d*^Porous NdF_3_ (Fig. 8a and b) as support.

^*e*^The commercial Ru/C catalyst (Fig. S6b and c). The quantitative analysis of the product was carried out using GC.

^*f*^ *Y* = yield of cyclohexane.

**Scheme 3:**
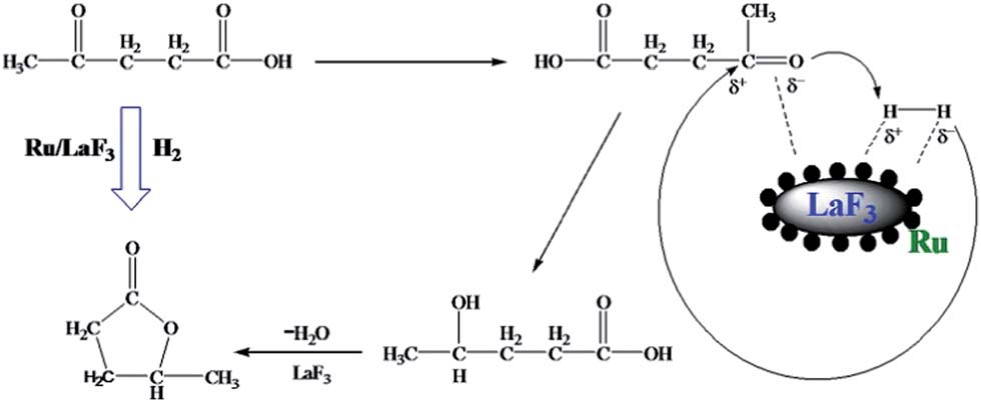
The reaction mechanism for LA hydrogenation to GVL.

LA hydrogenation to GVL is an important reaction in biomass utilization, and Ru is a very efficient catalyst for the reaction.^32^ In this work, we carried out the reaction catalyzed by the Ru/LaF_3_ prepared in this work and commercial Ru/C catalyst under solvent-free conditions, and the results are listed in Table 4. The dependence of the conversion on reaction time at some typical conditions is shown in Fig. S7.† The Ru/LaF_3_ had much higher activity than the commercial Ru/C and the supported Ru nanocatalysts reported by other researchers. The LaF_3_ interacts strongly with the carbonyl group by the Lewis acid–base interaction, which may be favourable to the reaction. In addition, an esterification reaction is feasible under acid and alkali conditions and the LaF_3_ as a Lewis acid can promote the esterification process. Therefore, the special morphology and structure, Lewis acid nature, and enhanced Lewis acidity by the defects of the LaF_3_ support are the main reasons for the extremely high activity and selectivity of the Ru/LaF_3_ catalyst for the reaction (Scheme 3).^33^

**Table 4:** Catalytic performances of the Ru/LaF_3_ and commercial Ru/C catalysts for LA hydrogenation to GVL^*a*^ ^,^ ^*f*^

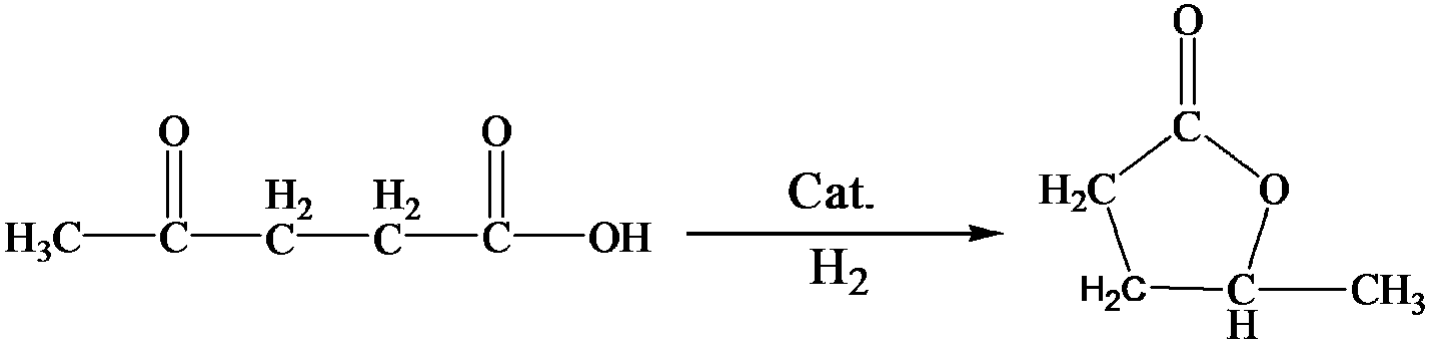						
Entry	Catalysts	*T* (°C)	*t* (h)	*C* (%)	*S* (%)	TOF^*b*^ (h^–1^)
1	Ru/LaF_3_ ^*c*^	130	0.5	>99	>99	4000
2	Ru/LaF_3_ ^*c*^	100	1.25	>99	>99	1600
3	Ru/LaF_3_ ^*c*^	70	3.5	>99	>99	571
4	Ru/LaF_3_ ^*c*^	40	9	>99	>99	222
5	Ru/C^*d*^	130	7	>99	>99	286
6	Ru/C^*d*^	100	18	>99	>99	111
7	Ru/Al_2_O_3_ ^*e*^	70	3	24	96	137

^*a*^Reaction conditions: 2 mL LA, LA/Ru (mol mol^–1^) = 2000, initial pressure of H_2_ was 6 MPa, solvent free.

^*b*^TOF was calculated as moles of converted LA per mole of Ru per hour.

^*c*^Porous LaF_3_ (Fig. 2a and b) as support.

^*d*^The commercial Ru/C catalyst (Fig. S6b and c).

^*e*^The catalyst reported in ref. 32. The quantitative analysis of the product was carried out by GC.

^*f*^ *C* = conversion of LA, *S* = selectivity of GVL.

## Conclusions

In summary, a protocol to synthesize mesoporous inorganic salts was proposed in this work, and mesoporous LaF_3_, NdF_3_ and YF_3_ were prepared successfully and were full of crystal defects. The catalytic activity of the mesoporous LaF_3_, NdF_3_ and YF_3_ particles could be more than 800 times higher than that of the commercial LaF_3_ without pores for the cyanosilylation reaction of benzaldehyde using trimethylsilyl cyanide. The catalytic activity of the supported catalysts Ru/LaF_3_ and Ru/NdF_3_, in which Ru nanocatalysts were supported on the mesoporous salt particles, was also extremely high for the hydrogenations of benzene to cyclohexane and LA to GVL. The abundant crystal defects in the mesoporous salt particles contributed significantly to the excellent catalytic performance of the catalysts. We believe that this method can also be used to fabricate some other mesoporous inorganic salts, which may have unique features in catalysis, extraction and fractionation, gas absorption, and other fields.

## Experimental

### Materials

OmimCl (purity > 99%) and N(Bu)_4_PF_6_ (purity > 99%) were purchased from the Centre of Green Chemistry and Catalysis, LICP, CAS. LaF_3_ (purity > 99.99%), La(NO_3_)_3_·6H_2_O (A. R. grade), Nd(NO_3_)_3_·6H_2_O (A. R. grade), Y(NO_3_)_3_·6H_2_O (A. R. grade), RuCl_3_·3H_2_O (Ru ≥ 37%), benzene (A. R. grade), ethanol (A. R. grade), cyclohexane (A. R. grade), toluene (A. R. grade), and *n*-butyl alcohol (A. R. grade) were provided by Sinopharm Chemical Reagent Co., Ltd. Benzaldehyde (purity > 98%), trimethylsilyl cyanide (purity > 97%), H_3_BTC (purity > 99%), LA (purity > 99%) and GVL (purity > 99%) were obtained from J & K Scientific Ltd. NaBH_4_ (purity ≥ 98%) was provided by Alfa Aesar China (Tianjin) Co., Ltd. The commercial Ru/C catalyst was purchased from Baoji Ruike Corporation, China.

### The solubility of N(Bu)_4_PF_6_ in OmimCl

In the experiment, known amounts of N(Bu)_4_PF_6_ and OmimCl were mixed in a glass tube with an inner diameter of 10 mm, which were immersed in an oil bath of desired temperature. The total mass of the mixture was 5.0 g. The mixture was heated to 160 °C to obtain a homogeneous solution. Then the temperature was decreased slowly until the solution became cloudy, indicating that N(Bu)_4_PF_6_ began to precipitate. At this temperature, the mixture became clear again as the temperature was increased. The process was repeated five times to determine the saturation temperature.

### SAXS study

The apparatus and the procedures were similar to that used to study the microstructure of IL gels.^13*b*^ SAXS experiments were carried out at Beamline 1W2A at the Beijing Synchrotron Radiation Facility. The data were collected using a CCD detector (MAR) with a maximum resolution of 3450 × 3450 pixels. The wavelength of the X-ray was 1.54 Å, and the distance of the sample to detector was 1.596 m. For OmimCl/N(Bu)_4_PF_6_ samples, desired amounts of OmimCl and N(Bu)_4_PF_6_ were mixed in a glass tube at 160 °C to obtain a homogeneous solution. Then the glass tube was cooled to 85 °C. In a typical experiment, some sample was added into the sample cell with a heating unit around the cell, and the X-ray scattering data were recorded. For the LaF_3_ samples, the sample was smeared on the sample cell at room temperature, and the other procedures were similar. The 2-D SAXS images were obtained from the detector and then transformed into the profiles of intensity (*I*) *vs.* wavevector (*q*) using the software SAXS Data Pre-process V2.0.0. The pair-distance distribution function *p*(r) was obtained from SAXS data by using an Irena tool suite within the Igor pro and Gnom application software. The fractal dimension *D* of LaF_3_ materials was calculated and analyzed using the method reported.^22^

### Synthesis of the porous salt particles

We describe mainly the procedures to synthesize LaF_3_ because those to synthesize NdF_3_ and YF_3_ were similar. In a typical experiment, desired amounts of OmimCl and N(Bu)_4_PF_6_ were mixed in a conical flask of 50 mL at 160 °C until the mixture became a homogeneous solution. The total mass of the mixture was 18 g. Then 1.5 mmol of La(NO_3_)_3_ was added to the system under vigorous stirring, and then the mixture was cooled to 85 °C in 10 min. The system was maintained at this temperature for 72 h. The obtained mixture containing the materials was mixed with 50 mL acetone and then centrifuged with a centrifugal speed of 5000 rpm. The obtained LaF_3_ was washed with acetone 10 times and dried in a vacuum oven at 40 °C for 24 h. To synthesize NdF_3_ or YF_3_, the procedures were the same. The main difference was that Nd(NO_3_)_3_ or Y(NO_3_)_3_ were used, instead of La(NO_3_)_3_.

### Synthesis of the La-MOF

The procedure for synthesizing the La-MOF was similar to that used previously.^28^ In the experiments, 18 g of OmimCl and water were mixed in a conical flask of 50 mL, and the molar fraction of water in the solution was 0.5. Then 1.5 mmol of La(NO_3_)_3_·*n*H_2_O and 1.5 mmol H_3_BTC were added to the conical flask. The system was maintained at 85 °C for 72 h under stirring. The obtained mixture containing the materials was centrifuged with a centrifugal speed of 5000 rpm. The obtained La-MOF was washed with acetone 10 times and dried in a vacuum oven at 40 °C for 24 h.

### Ru/LaF_3_ and Ru/NdF_3_ synthesis

We describe the procedure to synthesize Ru/LaF_3_ because that for the synthesis of Ru/NdF_3_ was similar. In a typical experiment, 20 mL RuCl_3_ aqueous solution (1 mmol) was added into a 50 mL flask. Then 0.2 g LaF_3_ was introduced into the flask and dispersed by ultrasound. The solution was stirred for 0.5 h. Then, 10 mL NaBH_4_ aqueous solution (20 mmol) was added into the mixture slowly until the Ru^3+^ was completely reduced. The Ru/LaF_3_ was washed with water five times and dried at 40 °C for 24 h under vacuum.

### Characterization

X-Ray diffraction (XRD) analysis of the samples was performed on an X-ray diffractometer (Model D/MAX2500, Rigaka) with Cu-Kα radiation, and the scan speed was 5° min^–1^. The morphologies of the products were characterized using a HITACHI S-4800 scanning electron microscope (SEM) and a JEOL-1010 transmission electron microscope operated at 100 kV. The crystal structure and SAED pattern were characterized using a TEM JEOL-2100F. The porosity properties of the materials were obtained from nitrogen adsorption–desorption isotherms using a Micromeritics ASAP 2020M system, and then calculated from the Barrett–Joyner–Halenda method. The Ru loading in the Ru/LaF_3_ catalyst and Ru/NdF_3_ was determined using the ICP-AES method (VISTA-MPX).

### Cyanosilylation of benzaldehyde

To carry out the reaction, 2 mL benzaldehyde and a certain amount of LaF_3_ were placed into a 25 mL glass vial, which was immersed in a constant-temperature water bath of 50 °C. The mixture was stirred (800 rpm) under a N_2_ atmosphere, and then TMSCN (another reactant) was added and the reaction began.^27^ After a suitable reaction time, the reaction mixture was centrifuged to precipitate the LaF_3_ particles. The reaction mixture was analyzed using a gas chromatograph (GC, HP 4890) equipped with a flame ionization detector (FID), and toluene was used as the internal standard. The identification of the products and reactants was done using a GC-MS (SHIMADZU-QP2010) and the ^1^H NMR spectra were recorded on a Bruker Avance III 400 HD spectrometer in CDCl_3_ with TMS as an internal standard, as well as by comparing the retention times to respective standards in GC traces.

### Benzene hydrogenation

The solvent free hydrogenation of benzene to cyclohexane was carried out in a 20 mL stainless steel batch reactor, which was similar to that used previously.^31*b*^ In the experiment, 2 mL benzene and a desired amount of catalyst were added into the reactor. The reactor was immersed in a water bath of desired temperature, and the reaction was started. Then H_2_ was introduced into the system at 4 MPa and the pressure was maintained during the reaction. The reaction mixture was cooled in ice-water and H_2_ was released after 10 minutes. The procedures for products analysis were similar to those described above. In the reuse experiments, the catalyst was washed with ethanol (3 × 5 mL) and was used for the next run after drying at 40 °C under vacuum.

### LA hydrogenation

The solvent free hydrogenation of LA to GVL was carried out in a 20 mL stainless steel batch reactor, which was the same as that for the hydrogenation of benzene described above. In the experiment, 2 mL LA and a certain amount of catalyst were added into the reactor. The reactor was immersed in an oil bath of desired temperature. Then H_2_ was introduced into the system at 6 MPa and the stirrer was started. The reaction mixture was cooled in ice-water for 10 minutes and H_2_ was released. The procedures for product analysis were similar to those described above. Differently, the internal standard for this reaction was *n*-butyl alcohol.
