# Supplementary material for: Mesoporous inorganic salts with crystal defects: unusual catalysts and catalyst supports
Source: Chem Sci. 2015 Jan 6;6(3):1668–75. doi: 10.1039/c4sc03736g (PMC5639790; doi:10.1039/c4sc03736g)
Supplement: Supplementary file 1 [file SC-006-C4SC03736G-s001.pdf]

Electronic Supplementary Material (ESI) for Chemical Science.
This journal is © The Royal Society of Chemistry 2015

Supplementary Information

Mesoporous Inorganic Salts with Crystal Defects: Unusual Catalysts and Catalyst Supports

Xinchen Kang,[a] Wenting Shang,[a] Qinggong Zhu,[a] Jianling Zhang,[a] Tao Jiang,*[a] Buxing Han,*[a] Zhonghua Wu,[b] Zhihong Li,[b] Xueqing Xing[b]

Results and Discussion

$$PF_6^- + H_2O + La^{3+} \rightarrow PF_5 \cdot H_2O + LaF_3$$

Scheme S1. The reaction equation of synthesizing LaF_3 using $N(Bu)_4PF_6$ and $La(NO_3)_3$.

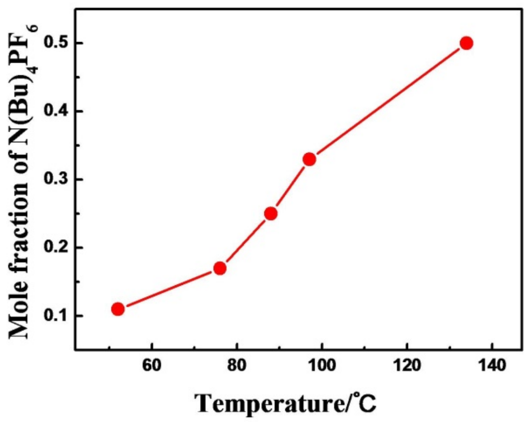

Figure S1. The solubility (mole fraction) of $N(Bu)_4PF_6$ in OmimCl at different temperatures.

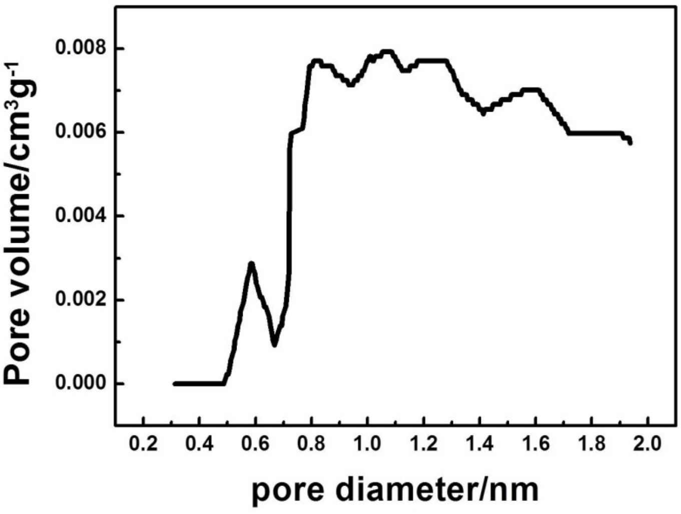

Figure S2. Pore size distribution of the micropores of LaF_3 synthesized at x_2= 0.5.

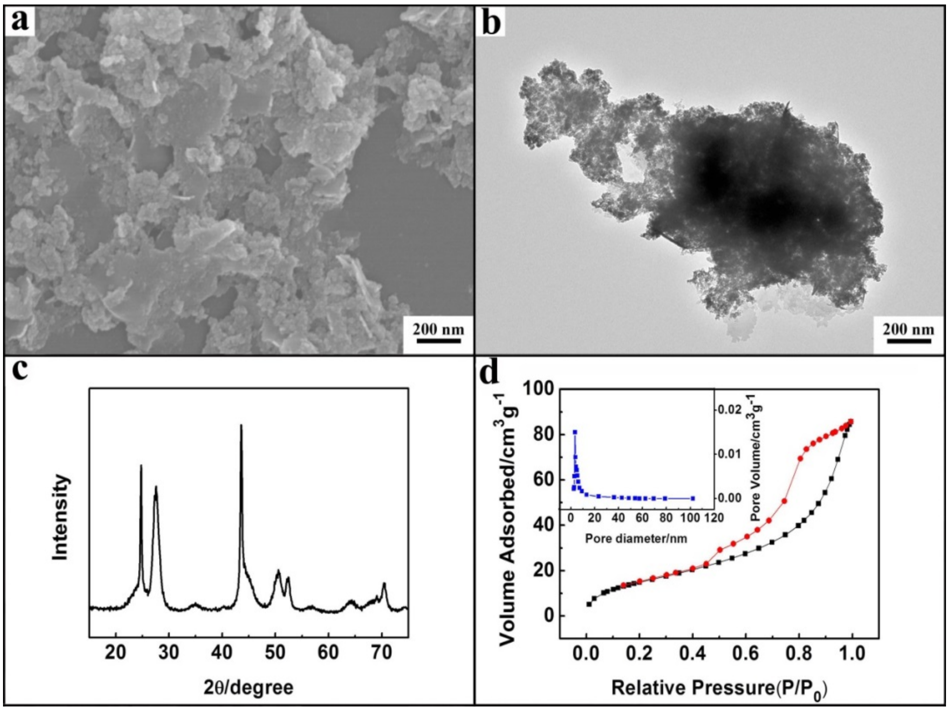

Figure S3. Characterizations of the LaF_3 particles synthesized at x_2=0.17: (a) SEM image; (b) TEM image; (c) XRD pattern; (d) N_2 adsorption/desorption isotherm and pore size distribution (the inset).

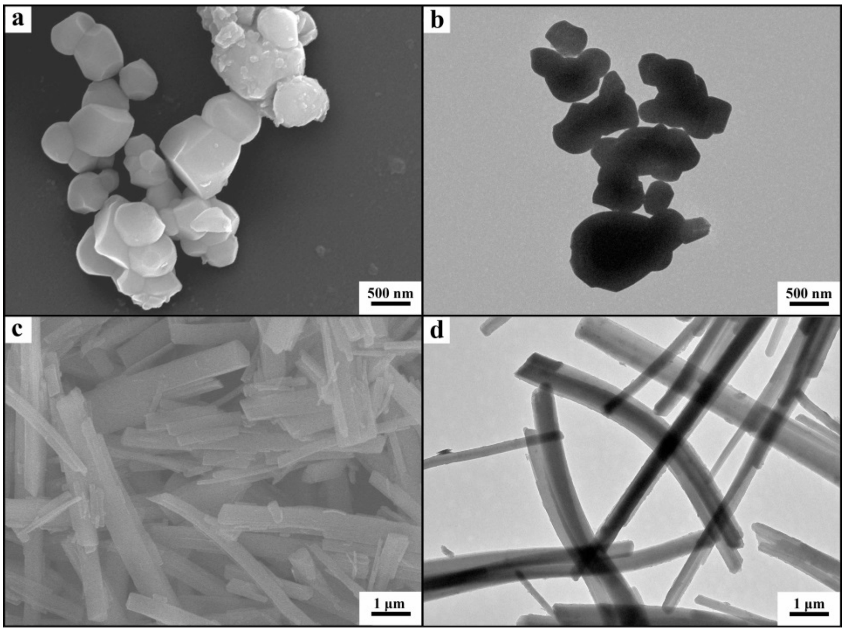

Figure S4. SEM and TEM images of commercial LaF_3 (a-b) and La-BTC MOF (c-d) synthesized in this work.

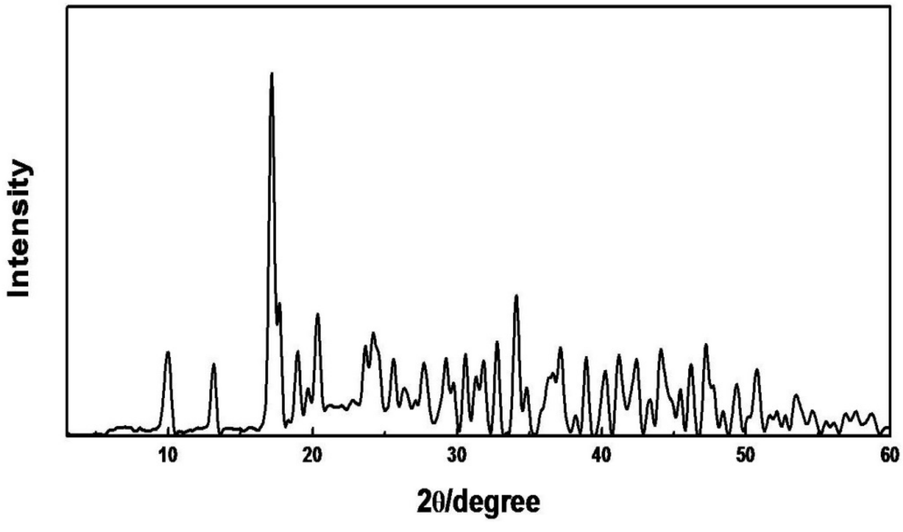

Figure S5. XRD pattern of La-BTC MOF synthesized in this work.

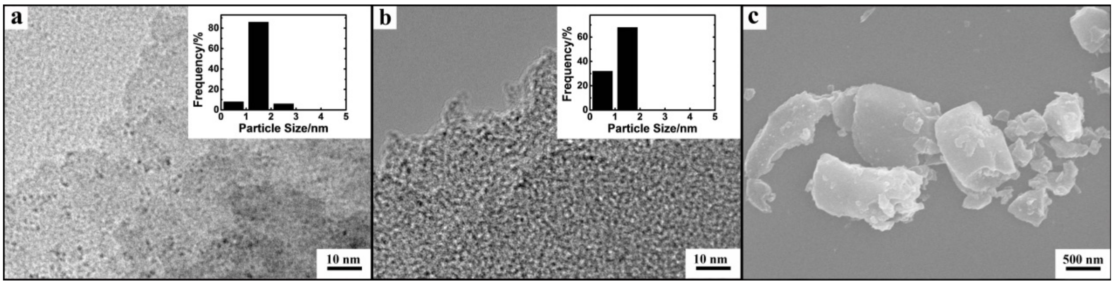

Figure S6. The TEM and SEM images of Ru/LaF_3 and commercial Ru/C catalyst: (a) TEM image of Ru/LaF_3 catalyst with 1.0 wt% Ru; (b) TEM image of commercial Ru/C catalyst with 5.0 wt% Ru; (c) SEM image of commercial Ru/C catalyst with 5.0 wt% Ru. The insets in (a and b) show the size distribution of Ru particles.

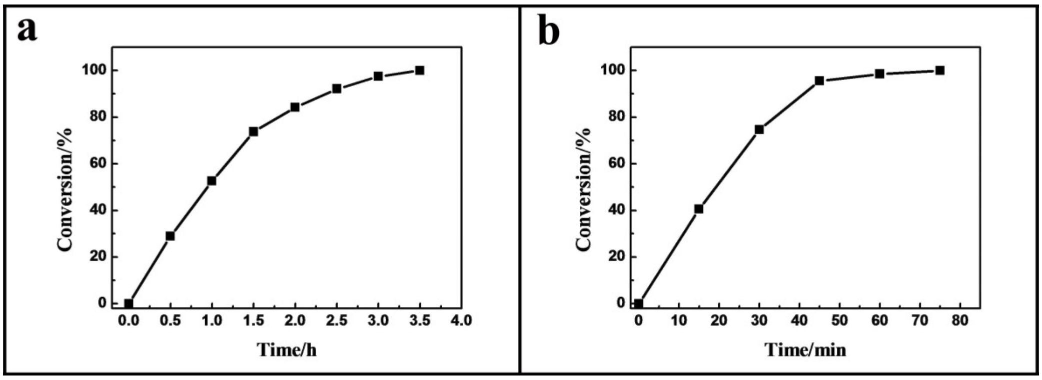

Figure S7. The dependence of the conversion of LA on reaction time at 70 ^oC (a) and 100 ^oC (b). Other conditions were the same as that for entries 2 and 3 in Table 4.
